# “Addressing rising dengue burden in Pakistan: is it time to consider dengue vaccination?”

**DOI:** 10.1097/MS9.0000000000004072

**Published:** 2025-11-07

**Authors:** Muhammad Momin Khan, Manaal Shakir, Sahir Bansari, Bushra Azam Hashmi, Farina Fatima Siddiqui, Muhammad Idrees, Shahzad Hussain, Ajay Pandeya

**Affiliations:** a Department of Internal Medicine, Liaquat University Hospital, Hyderabad, Sindh, Pakistan; b Department of Neurology, Punjab Institute of Neurosciences, Lahore, Punjab, Pakistan; c Department of Internal Medicine, Bilawal Medical College, Jamshoro, Sindh, Pakistan; d Department of Internal Medicine, Lahore General Hospital, Lahore, Punjab, Pakistan; e Department of Medicine, KIST Medical College, Lalitpur, Nepal

*Dear Editor,*

## Introduction: background on dengue and its trends in Pakistan

Dengue virus (DENV 1–4), a member of the *Flaviviridae* family, is predominantly found in tropical and subtropical regions, including Southeast Asia, India, and Pakistan. Its transmission is driven by outbreaks that are increasingly fueled by rapid urbanization, climate change, and expanding mosquito vector populations[1]. It carries a significant burden worldwide with 13 million cases and 10 000 fatalities reported in 2024, though the actual number is likely higher due to underreporting in low- and middle-income countries[2]. Usually, the disease presents with sudden high-grade fever, headache, muscle and joint pain, rash, and nausea, which may progress to hemorrhage, multiorgan failure, and septic shock[2]. In line with current best practices for transparency and accountability in scientific reporting, this manuscript adheres to the TITAN guidelines for responsible disclosure of AI assistance in research and writing[3].

## Burden analysis: national and regional statistics

**Figure 1.**
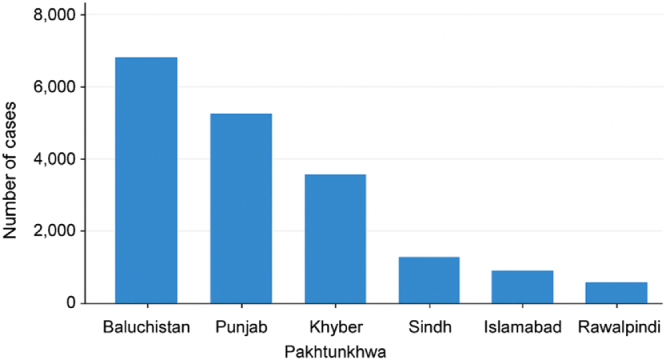
Dengue cases by province in Pakistan, 2024.

**Figure 2.**
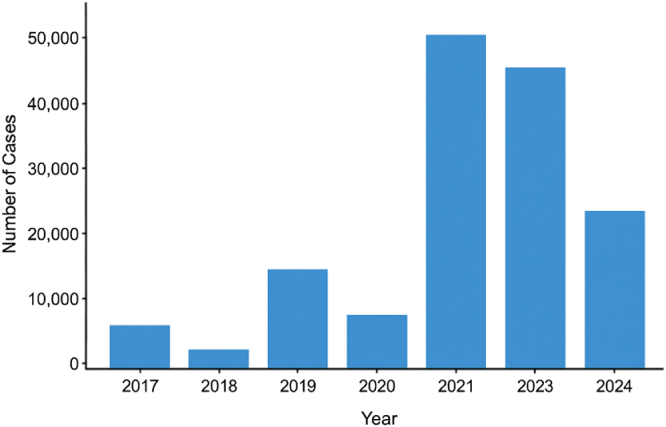
Dengue cases in Pakistan (2017–2024).

Regionally, Bangladesh recorded 97 159 dengue cases and 532 fatalities in 2024, while India reported 186 567 cases and 160 deaths as of October 2024, with grim projections for 2025 estimating up to 280 000 cases[4]. In contrast, Iran reported fewer cases at 221, primarily attributed to imports from neighboring countries[5]. As of December 2024, Pakistan has reported 20 000 dengue fever cases, including a sharp rise of nearly 3000 cases in a single week in June[6]. With the regional calibration of dengue cases in Pakistan, the province of Balochistan reported the highest case count at 6958, followed by Punjab (5405), Khyber Pakhtunkhwa (3649), and Sindh (1167)[6]. Rawalpindi emerged as the most affected urban center in Pakistan, reporting 2143 cases, while the capital city, Islamabad, recorded 3754 cases[7] (Figure 1). A significant rise in the incidence of dengue has been experienced over the last 5 years in Pakistan, as the country harbored 48 906 new dengue cases and 183 deaths in 2021, followed by 41 746 cases and 62 fatalities in 2022[8]. In the year 2023, the country encountered 20 072 new cases[8]. Previously, 22 938 cases in 2017, over 3200 in 2018, 24 547 in 2019, and 3442 cases in 2020 were reported[9] (Figure 2). These fluctuating trends underscore the ongoing and rapidly increasing public health challenges imposed by the virus in the country.

## Current control measures and limitations

Currently, no specific antiviral treatments are available for dengue fever. The approach to clinical management is primarily centered on supportive care by attenuating the inflammatory responses using analgesics, antipyretics, and intravenous fluids[10]. The World Health Organization advocates the implementation of Integrated Vector Management (IVM) to control mosquito populations[11]. IVM should focus on eliminating breeding sites, reducing vector populations, and minimizing human exposure through larval and adult mosquito control, environmental management, and chemical interventions. Strengthening vector control efforts in Pakistan requires the integration of dengue vaccines to enhance population immunity. Vaccine development represents a critical strategy to reduce disease incidence and severity, complement existing vector control measures, and substantially decrease the burden on the healthcare system[12]. In endemic regions, where outbreaks already strain the resources, an effective vaccine can curb transmission, lower morbidity and mortality rates, and reduce economic losses associated with illness and vector control efforts.

## Vaccine landscape: trials and mechanisms

One of the major challenges in developing a dengue vaccine is the absence of reliable animal models for experimental studies and the unresolved issue of immune interference[13]. In the context of immunology, a tetravalent live attenuated vaccine should elicit independent monotypic immune responses for the four dengue serotypes; however, achieving such a balanced tetravalent immune response has proven to be highly complex. Live attenuated vaccines are the only type of vaccines to have advanced to phase 3 clinical trials[14]. The failure of inactivated vaccines to elicit a strong immune response has been postulated secondary to alterations in antigenic sites critical for initiating effective immunity (Supplemental Digital Content Table S1, available at: http://links.lww.com/JS9/F553)[15]. In addition to immunogenicity concerns, significant barriers to vaccine development include safety profiles, delivery logistics, production and stability limitations, variable efficacy, and cost-effectiveness[15].

The development of the chimeric yellow fever 17D-based tetravalent dengue vaccine, Dengvaxia® (CYD-TDV), involved 26 clinical trials with over 41 000 participants. Of these, more than half of the individuals, including 20 974 individuals aged 9–45, received at least one dose of the tetravalent formulation[16]. Akter *et al*[17] report in a phase 3 trial results that the vaccine’s efficacy varied based on age, serostatus, and dengue serotype, yet it demonstrated a population-level benefit. Recombinant DNA technology plays a crucial role in developing this chimeric vaccine.

Qdenga® (TAK-003), a live attenuated tetravalent dengue vaccine, has been developed using a DENV2 backbone[18], Tricou *et al* reported that over the follow-up period of 4.5 years, the cumulative efficacy was nearly 60%, in baseline seronegative participants, varied by serotype^[18,19]^. The vaccine showed better protection in individuals with prior dengue infections than in dengue-naïve individuals. Qdenga® induces antibody responses against all four dengue serotypes, with the highest levels against DENV2. Antibody levels are notably higher in individuals with prior dengue infection and remain above the cutoff for seropositivity in most individuals for several years after vaccination. While efficacy declined over time, it remained consistent in preventing severe, hospitalized dengue cases[20]. However, no defined serological correlate of protection has been established.

More recently, TV003, a dengue vaccine, has shown encouraging outcomes in clinical trials, achieving an efficacy of about 80%[21]. The trials reported strong immune responses to all four serotypes, with notable protection against DENV-1 and DENV-2, even among individuals without dengue exposure[21]. However, due to a limited number of cases for DENV-3 and DENV-4 in the trials, its effectiveness against these serotypes remains uncertain. Ongoing follow-up studies are expected to provide further clarity. As TV003 progresses through advanced development stages, it shows great potential as a valuable tool in combating dengue worldwide.

Despite advances in vaccine development, the feasibility of large-scale dengue vaccination programs faces important challenges. Regulatory approval processes, post-marketing surveillance, and the need for robust cold chain systems remain major hurdles in low- and middle-income countries such as Pakistan. Furthermore, prior controversies surrounding Dengvaxia®, including its association with an increased risk of severe dengue among seronegative recipients, which led to restrictions in its use and public mistrust in some countries, highlight the importance of cautious deployment and community engagement. These regulatory and social considerations must be addressed alongside scientific progress to ensure that future vaccines can be safely and effectively implemented in endemic regions.

## Pakistan’s readiness and challenges

Pakistan’s healthcare system is under significant strain due to inadequate infrastructure, compounded by overpopulation, poor sanitation, unregulated urbanization, and limited public health support. This fragility was starkly demonstrated during the widespread flooding of 2022, which damaged approximately 888 healthcare facilities and affected one-third of the country, further exacerbating an already critical resource shortage[22]. Pakistan has only six tertiary care hospital beds per 10 000 people, highlighting the immense pressure on its healthcare system[23]. Despite government-funded treatment, patients still incur an average hospitalization cost of Rs. 21 242 (USD 248), much higher than the average household income in Pakistan[24]. Efforts to control mosquito populations, the primary vector for the virus, have proven inadequate, underscoring the need for alternative strategies. Given the increasingly severe dengue outbreaks since 2011, controlling transmission through vector control alone is no longer sufficient. A dengue vaccine could significantly alleviate the burden on Pakistan’s healthcare system by reducing the number of severe cases requiring hospitalization, thereby easing pressure on the already strained infrastructure. Despite progress in vaccine development, these shortcomings highlight the need for novel vaccine candidates with improved safety, efficacy, and broad-spectrum coverage.

## Recommendations

To address these limitations, future research should prioritize novel vaccine platforms that elicit robust, long-lasting immunity across all four dengue serotypes while ensuring safety for seronegative individuals. RNA-based vaccines, leveraging advances in mRNA technology seen in COVID-19 vaccines, can induce strong humoral and cellular immune responses with enhanced stability and scalability. Additionally, nanoparticle-based vaccines, incorporating antigenic components from all four dengue serotypes, may provide a more consistent immune response. Exploring innovative adjuvant systems and delivery methods, such as viral-vectored and thermostable formulations, could further enhance vaccine efficacy and accessibility in endemic regions. By prioritizing research into RNA-based, nanoparticle, and novel adjuvant vaccine platforms, the global scientific community can accelerate progress toward the development of an effective and universally applicable dengue vaccine.

## Conclusion

In conclusion, dengue remains a pressing public health challenge in Pakistan and globally, with current control measures proving insufficient. Strengthening vaccine development, particularly through innovative mRNA and nanoparticle platforms, offers a promising path toward durable protection, reduced disease burden, and improved healthcare resilience in endemic regions.

## Data Availability

Not applicable.
